# Unbiased kinome profiling identifies key and novel mediators of chronic kidney disease in hyperlipidemic mice

**DOI:** 10.3389/fphys.2025.1684982

**Published:** 2025-10-02

**Authors:** Andrea Bonnin-Marquez, Sanne L. Maas, Melissa Corcini-Berndt, Emiel P. C. Van der Vorst

**Affiliations:** ^1^ Department of Internal Medicine I, Aachen-Maastricht Institute for Cardio-Renal Disease (AMICARE), and Institute for Molecular Cardiovascular Research (IMCAR), University Hospital Aachen, RWTH Aachen University, Aachen, Germany; ^2^ Institute for Cardiovascular Prevention (IPEK), Ludwig-Maximilians-Universität, Munich, Germany

**Keywords:** chronic kidney disease, hyperlipidemia, kinases, kinomics, cardiorenal

## Abstract

**Introduction:**

Chronic kidney disease (CKD) is a progressive condition associated with increased mortality and morbidity, placing a substantial burden on healthcare systems globally. CKD often coexists with cardiovascular disease (CVD), further complicating patient outcomes. This study investigates the kinomic profile of hyperlipidemic mice to understand the signaling mechanisms underlying CKD progression and its cardiovascular consequences.

**Methods:**

*Apoe* ^*−/−*^ mice were subjected to a Western-type diet, with or without adenine supplementation to induce CKD. Kinase activity was profiled using PamGene® assays on renal cortex samples collected at early (4 weeks) and late (12 weeks) stages of CKD.

**Results:**

It could be demonstrated that CKD led to significant increases in peptide phosphorylation related to both tyrosine and serine-threonine kinases, which were particularly pronounced in the late-stage model. Therefore, the kinase activity in the kidney increased upon CKD development in a CKD-stage-dependent manner. Notably, the activity of cyclin-dependent kinases (CDKs) was reduced at early disease stages but remained unaffected in late stages. Pathway analysis revealed stage-specific alterations in cell cycle regulation, inflammation, oxidative stress, lipid metabolism, and fibrosis pathways associated with kinase activity changes throughout disease progression.

**Discussion:**

These findings highlight critical kinases involved in CKD development and suggest their potential roles in mediating pathological processes such as inflammation and fibrosis. Targeting specific kinases may offer novel therapeutic strategies for mitigating CKD progression and its cardiovascular complications. Future research should explore the causal relationships between newly identified kinases and CKD development.

## 1 Introduction

Chronic kidney disease (CKD) is a complex, progressive disease with substantial consequences globally. CKD patients suffer from increased mortality and morbidity, and treatment, particularly in advanced stages, poses a significant economic burden (Foreman et al., 2018; Jha et al., 2023; Levin et al., 2017; Stewart et al., 2024). According to current international guidelines, CKD is defined as abnormalities in kidney function or structure persisting for at least 3 months, with consequences for patient health (Kidney Disease: Improving Global Outcomes CKDWG, 2024; Stevens et al., 2013). Disease severity is classified based on glomerular filtration rate (GFR), albuminuria, and underlying cause (Kidney Disease: Improving Global Outcomes CKDWG, 2024; Stevens et al., 2013). These alterations to the function and structure of the kidney are irreversible and, in the early stages, often asymptomatic, leading to late diagnoses (Webster et al., 2017). Moreover, there is no effective cure, and patients who progress to end-stage kidney disease (ESKD) require kidney replacement therapy, either via dialysis or kidney transplant (Webster et al., 2017). Despite advancements in diagnostic guidelines and treatments, CKD remains underdiagnosed and undertreated worldwide (Stewart et al., 2024). In addition, CKD has not been included in the World Health Organization’s list of major drivers of non-communicable disease-related early mortality, limiting both resource allocation and recognition of the disease’s impact (Francis et al., 2024). Consequently, CKD-related mortality has continued to rise over the last decades, and the disease is projected to become the fifth leading cause of years of life lost globally by 2040 (Foreman et al., 2018; Stewart et al., 2024; Collaboration, 2020).

In addition to its direct health burden, CKD patients are at an increased risk for cardiovascular disease (CVD), with the majority of CKD patients succumbing to cardiovascular events rather than from ESKD (Foreman et al., 2018; Kidney Disease: Improving Global Outcomes CKDWG, 2024; Webster et al., 2017; Vaduganathan et al., 2022). Furthermore, CKD can also occur as a comorbidity of various pathologies, including diabetes, hypertension, heart failure, and atherosclerosis (Jankowski et al., 2021; Marx-Schutt et al., 2025; Noels et al., 2025; Rangaswam et al., 2019). Patients with such comorbidities face particularly poor prognoses, with reduced survival and diminished quality of life (Fraser et al., 2015). Notably, CKD patients exhibit a markedly increased risk for atherosclerosis, which is the main underlying cause of CVD (Jankowski et al., 2021; Marx-Schutt et al., 2025). CKD and CVD share various pathological mechanisms, including inflammation, oxidative stress, fibrosis (Kumar et al., 2019), and hyperlipidemia (Visconti et al., 2016). The most common dyslipidemia pattern in CKD patients consists of hypertriglyceridemia and reduced high-density lipoprotein levels (Reiss et al., 2015). In recent years, triglycerides and triglyceride-rich lipoproteins, which are not the typical target of lipid-lowering therapies, have gained more recognition as a causal factor in atherosclerosis and as an independent risk factor for cardiovascular complications (Xu et al., 2024). Despite these well-established mechanistic links between CVD and CKD, the field of cardiorenal research only began to expand from the early 2000s (Lv et al., 2023). As a result, a deeper understanding is still needed to uncover additional contributors and critical regulatory pathways involved in CKD and CKD-induced atherosclerosis.

Major regulators of cellular processes relevant to CKD are protein kinases, which mediate signal transduction via phosphorylation (Ubersax and Ferrell, 2007), resulting in diverse functional effects on cellular activity (Manning et al., 2002). Kinases are commonly classified into two main groups, tyrosine-specific kinases and serine/threonine-specific kinases (Ubersax and Ferrell, 2007). Kinases are one of the largest gene families, making up approximately 2% of the genome, and are estimated to phosphorylate about 30% of all cellular proteins on at least one residue (Ubersax and Ferrell, 2007), underscoring their critical biological importance.

Given this central role, we decided to employ a kinomics approach to explore the signaling mechanisms at play in CKD. More specifically, we studied the kinomic profile of the kidneys of hyperlipidemic mice with or without CKD, as hyperlipidemia is a major characteristic of both CKD and atherosclerosis, and particularly a driver of CKD-induced atherosclerosis. In addition, we evaluate kinase activity at both early and advanced stages of disease to observe changes associated with disease progression.

## 2 Materials and methods

### 2.1 Mice

All mice were apolipoprotein E (*Apoe)* ^*−/−*^ and bred on a C57/Bl6 background for more than 10 generations (Jackson Laboratory; Strain#: 002052). Starting at 8 weeks of age, mice were fed a Western-type diet (WTD) containing 21% fat and 0.15%–0.20% cholesterol (Altromin 132010, Sniff TD88137), supplemented with or without 0.3% adenine for 2 weeks to induce CKD. Subsequently, the mice were fed a WTD with or without 0.15% adenine for another 2 or 10 weeks to maintain CKD and stimulate CKD-induced hyperlipidemia.

To verify CKD induction, mice were placed in individual metabolic cages overnight to collect urine at baseline and at the end of the 4 or 12-week feeding period. Blood samples were collected at the indicated time points by retro-orbital bleeding after inhalational anesthesia using isoflurane and subsequently processed to obtain either serum or plasma. For functional renal analyses, urea, creatinine, and total protein levels were measured in urine and serum using a clinical renal test analyzer according to the manufacturer’s instructions. Plasma cholesterol and triglyceride concentrations were measured by using enzymatic assays (Roche diagnostics) following the manufacturer’s protocol.

Euthanasia was performed under deep, non-antagonisable anaesthesia (10 mg/kg xylazine and 100 mg/kg ketamine) with subsequent blood collection via retro-orbital puncture using anticoagulant-coated capillaries. All animal studies performed were approved by the local ethical committee (Landesamt für Natur, Umwelt und Verbraucherschutz Nordrhein-Westfalen, Germany, approval number 81-02.04.2019.A364). All procedures are in line with the guidelines from Directive 2010/63/EU of the European Parliament on the protection of animals used for scientific purposes.

### 2.2 Kinase activity profiling - Analysis

Snap-frozen renal cortex pieces (10 mg) were thawed on ice and lysed in M-PER™ Mammalian Protein Extraction Reagent (ThermoFisher Scientific) containing 1% Halt™ Protease Inhibitor Cocktail (ThermoFisher Scientific) and 1% Halt™ Phosphatase Inhibitor Cocktail (ThermoFisher Scientific) for 10 min. The tissue was homogenized with a metal bead in the TissueLyser LT (Qiagen) for 7 min at 50 Hz, after which the lysates were centrifuged at 15.000 x g for 15 min at 4°C. Protein amounts were measured using the Nanodrop One Microvolume UV-Vis Spectrophotometer (ThermoFisher Scientific).

The PamChip® peptide tyrosine kinase and Ser/Thr Kinase assay microarray systems were used on the PamStation®12 (PamGene International) to define the profiles of Tyrosine kinase (PTK) and Serine-Threonine kinase (STK), respectively. The PTK-PamChip® includes 196, and the STK-PamChip® array contains 144 individual phospho-sites. For PTK and STK activity profiling, all reagents were used by PamGene International B.V.

In the PTK assay, 10.0 μg of protein (n = 4 per condition) was used following the standard protocol provided by PamGene. Lysates were prepared by adding PTK Basic Mix, consisting of 4 μL of 10x protein PTK reaction buffer (PK), 0.4 μL of 100x BSA, 0.4 μL of 1 M dithiothreitol (DTT) solution, 4 μL of 10x PTK additive, 4 μL of 4 mM ATP, and 0.6 μL of monoclonal anti-phosphotyrosine FITC-conjugated detection antibody (clone PY20). Distilled water was added to bring the total volume to 40 μL. Before loading the samples, a blocking step was performed by applying 30 μL of a 2% BSA solution to the center of each array, followed by a wash with PTK solution for PamChip® preprocessing. Subsequently, 40 μL of the PTK Basic Mix was added to each array of the PamChips®. The microarray assays were then run for a total of 94 cycles on the PamStation®12. An image was recorded using a CCD camera at kinetic read cycles 32–93, with exposure times of 10, 50, and 200 ms, and an end-level read cycle at 10, 20, 50, 100, and 200 ms. Spot intensities at each time point were quantified and corrected for local background using BioNavigator software version 6.3 (PamGene International, ‘s-Hertogenbosch, The Netherlands).

In the STK assay, phosphorylated Ser/Thr residues were detected using 2.0 μg of protein per sample (n = 4 per condition) and 400 μM ATP, combined with the STK Basic Mix. The Basic mix consisted of 1× protein PTK reaction buffer (PK), 1x bovine serum albumin (BSA), and 1x STK antibody mix. Before loading the samples, a blocking step was performed using 30 µL of 2% BSA, followed by washing with PTK solution for preprocessing. Subsequently, 40 µL of STK Basic Mix was applied to each array. Samples were incubated for one hour at 30°C, during which the samples were actively pumped back and forth through the porous material to maximize binding kinetics and minimize assay time. Phosphorylation events were detected using a FITC-conjugated antibody. Imaging was done using an LED-based imaging system, and the spot intensities at each time point were quantified and corrected for local background using the BioNavigator software version 6.3 (PamGene International, ‘s-Hertogenbosch, The Netherlands).

### 2.3 Kinase activity profiling - Visualization

The distribution of the phosphorylated peptides is shown in the volcano plots created with the R package EnhancedVolcano v1.26.0 (Blighe et al., 2025). Blue dots depict peptides with an adjusted P value < 0.05.

A principal component analysis (PCA) was performed with the help of the R package stats V4.3.1 (Team, 2013), depicting the singular peptide decomposition, examining the covariances/correlations between the samples. Results were visualized as two-dimensional PCA plots generated with ggplot2 (Wickham, 2016), enabling the identification of sample clustering patterns, variability, and potential outliers.

To classify and visualize kinases identified in the assay, a kinase coral tree was generated using the Coral web tool (http://phanstiel-lab.med.unc.edu/CORAL). This approach organizes kinases based on their evolutionary and functional relationships into families and subfamilies, as defined by the Human Kinome Project classification system. Input data included the list of kinases detected as active in the PTK or STK PamChip® assays. Visualization parameters were set to reflect relative kinases; node/branch colors represent mean kinase statistics (<0.05), and node sizes represent the median final scores (>1.2). The hierarchical representation in the coral tree allowed the identification of family-wide trends, highlighting condition-specific kinase activation patterns, and supporting the functional interpretation of the kinase profiles.

Over-representation analysis (ORA) was performed for kinases with significant differences compared to control samples (median final scores >1.2 and adjusted p-value <0.05) using the Gene Ontology, Kyoto Encyclopedia of Genes and Genomes (KEGG), and WikiPathway databases. ORA was conducted using the R packages clusterProfiler v4.8.1 (Wu et al., 2021), and the data was visualized with the R package ggplot2 v3.4.2 (Wickham, 2016).

### 2.4 Statistical analysis

Statistical analyses were performed with GraphPad Prism v.10 (GraphPad Software, LLC). Gaussian distribution was tested using the D’Agostino & Pearson test, after which the Mann-Whitney U test was used to analyze the data. *P < 0.05; ****P < 0.0001.

## 3 Results

### 3.1 CKD increases peptide phosphorylation in the kidneys of hyperlipidemic mice.

**FIGURE 1:**
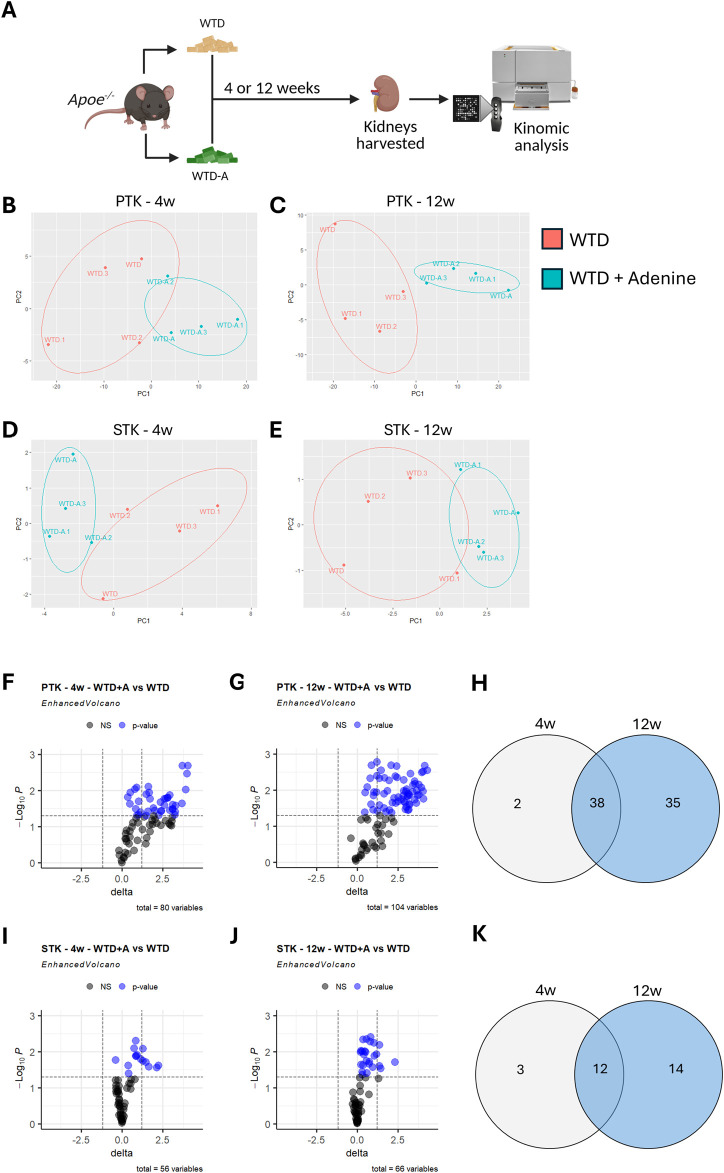
Peptide phosphorylation is increased in hyperlipidemic mice with CKD and upon disease progression. **(A)** Schematic diagram of the study design. **(B–E)** Principal component analysis (PCA) depicting the variance in peptide phosphorylation profiles in kidney lysates from control (WTD) and CKD mice (WTD + Adenine) for **(B)** PTK-related peptides after 4 weeks of diet, **(C)** PTK-related peptides after 12 weeks of diet, **(D)** STK-related peptides after 4 weeks of diet, or **(E)** STK-related peptides after 12 weeks of diet. **(F,G)** Volcano plots displaying differentially phosphorylated PTK-related peptides. Blue dots represent significant differential phosphorylation of peptides between the two groups (p < 0.05). Total variables reflect the number of peptides with measurable amounts of phosphorylation. **(H)** Venn diagram showing the overlap and unique sets of differentially phosphorylated PTK-related peptides in both early (4 weeks) and late (12 weeks) stages of CKD. **(I,J)** Volcano plots displaying differentially phosphorylated STK-related peptides. Blue dots represent significant differential phosphorylation of peptides between the two groups (p < 0.05). Total variables reflect the number of peptides with measurable amounts of phosphorylation. **(K)** Venn diagram summarizing differentially phosphorylated STK-related peptides shared or unique between early- and late-stage CKD. All data represent four biological replicates per condition.

*Apoe* ^*−/−*^ mice were fed with a pro-atherogenic Western-type diet (WTD) to induce hyperlipidemia, or a WTD enriched with adenine (WTD-A) to induce CKD in hyperlipidemic mice, for either 4 weeks (early-stage) or 12 weeks (late-stage). Subsequently, the renal cortex was collected to compare the kinomic profiles of hyperlipidemic mice with or without CKD (Figure 1A). Serum urea and creatinine levels significantly increased in WTD-A-fed mice, while urine urea and creatinine decreased, coinciding with an increased amount of urine protein, suggesting a successful induction of kidney damage (Supplementary Figures S1A-F, S2A-F). Circulating cholesterol and triglyceride levels did not change, while the body weight was slightly reduced in WTD-A-fed mice, again aligning with an induction of CKD in these mice (Supplementary Figures S1G-I, 2G-I). Kinase activity was measured with the PamGene® platform, revealing specific peptide phosphorylation as a proxy for kinase activity. Apparent differences in peptide phosphorylation patterns are observed between WTD-A and WTD-fed mice at both early- and late-stages of CKD (Figures 1B–E; Supplementary Tables 1-4). Particularly for the PTK, the magnitude of these differences increases upon disease progression, reaching its peak in the late-stage CKD model (Figures 1B,C). Further analysis reveals a significant increase in phosphorylation of peptides related to tyrosine kinases, with changes being more pronounced at 12 weeks than at 4 weeks (Figures 1F,G). Notably, 38 out of the total 75 differentially phosphorylated peptides are upregulated in both early and late stages, indicating substantial overlap (Figure 1H). Nevertheless, disease progression contributes to the phosphorylation of 35 additional peptides exclusively in the late-stage CKD group (Figure 1H).

A similar, although less pronounced, pattern is observed for peptides related to serine-threonine kinases (Figure 1I-K). For these peptides, 12 out of the total 29 differentially phosphorylated peptides overlap between the early- and late-stages of CKD, while 14 additional peptides are phosphorylated only in the late-stage (12 weeks), compared to the early-stage (4 weeks) CKD model (Figure 1K).

Overall, CKD in hyperlipidemic mice results in an increased phosphorylation of peptides related to both tyrosine and serine-threonine kinases in the kidney. Furthermore, the amount of phosphorylation increases with disease progression.

### 3.2 Differential kinase activity in kidneys of hyperlipidemic mice upon CKD development.

**FIGURE 2:**
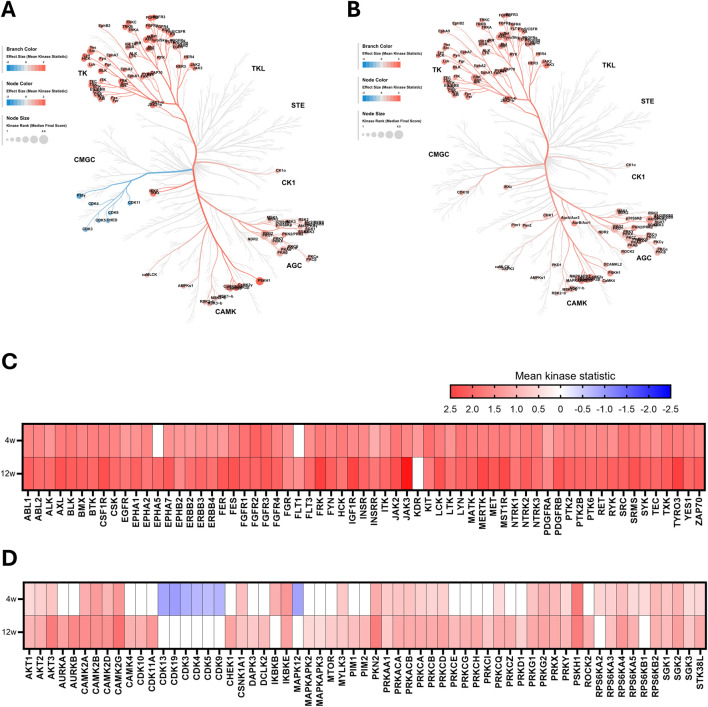
Overall kinase activity is elevated in hyperlipidemic mice with CKD. **(A,B)** Kinome coral trees depicting kinase rank (node size) and the effect size (node and branch color) of significantly differentially regulated kinases in kidney lysates after **(A)** 4 weeks and **(B)** 12 weeks of diet. Both STK and PTK are represented. **(C,D)** Heatmaps showing effect size (mean kinase statistic) for **(C)** PTK and **(D)** STK after 4 and 12 weeks of diet, sorted alphabetically. All data represent four biological replicates per condition.

We deployed an Upstream Kinase Analysis to determine which kinases are responsible for the differentially phosphorylated peptides observed in CKD. In line with the observed changes in peptide phosphorylation patterns, tyrosine kinase (TK) activity is strongly increased in mice fed with WTD-A, compared to WTD-fed mice (Figures 2A,B). Furthermore, while overall the activity of the same tyrosine kinases is significantly increased upon CKD development, kinases are slightly more active in the late-stage CKD model (12w) compared to the early-stage model (4w) (Figure 2C). A similar trend is observed for serine-threonine kinases, as the kinase activity is increased more in the late-stage CKD model (12w) compared to the early-stage model (4w) (Figure 2D).

Among all predicted kinases, Janus kinase 3 (JAK3) emerges as the most highly upregulated tyrosine kinase, particularly in late-stage CKD (Figure 2C). JAK3 is well known for its key role in lymphocyte homeostasis, proliferation, and activation (Borie et al., 2003). The most upregulated serine-threonine kinase is calcium/calmodulin-dependent protein kinase II gamma (CAMK2G) (Figure 2D). This kinase is known to modulate various processes, including calcium homeostasis, cell cycle, cytoskeletal organization, and a regulatory role in the inflammatory signaling through its function as an intracellular calcium sensor (Junho et al., 2020).

Interestingly, despite the general trend of increased kinase activity in mice with CKD, compared to mice without CKD, there is a distinct cluster of kinases that is strongly downregulated in the early-stage model (4w). This group includes several cyclin-dependent kinases (CDKs), best known for their critical role in cell cycle and gene transcription regulation (Malumbres, 2014), as well as mitogen-activated protein kinase 12 (MAPK12, also known as p38γ), which is known to play a role in inflammatory and metabolic pathways (Qi and Chen, 2023) (Figure 2D).

### 3.3 Cell cycle-related pathways in the kidney are affected by CKD development.

Pathway analysis was performed using ORA to understand the complex interplay of significantly differentially activated kinases. Given that CDKs showed a clear and distinct downregulation compared to other kinases, we first examined pathways in which these kinases are involved. As expected, these kinases are primarily associated with cell cycle regulation, a process of particular interest in CKD due to its role in maladaptive cell repair and promoting a pro-fibrotic phenotype.

**FIGURE 3:**
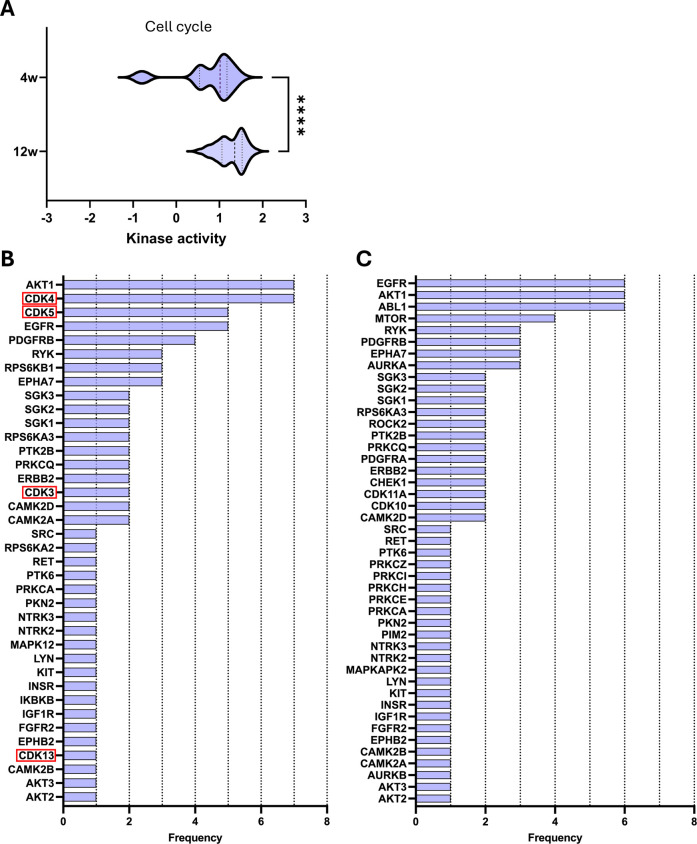
Key kinases play a significant role in selected cell cycle pathways during CKD development. **(A)** Violin plot of kinase activity in cell cycle-related pathways. Kinase activity is reflected by the mean kinase statistic of each kinase involved in these pathways. **(B,C)** Bar graphs of kinase frequency of kinase involvement across cell cycle pathways after **(B)** 4 weeks and **(C)** 12 weeks of diet. Downregulated kinases are highlighted with a red box. See Table 1 for all pathways (e.g., a frequency of 1 denotes that the kinase is involved in one pathway, while a frequency of 5 denotes that the kinase is involved in five distinctive pathways). Statistical significance was assessed by the Mann-Whitney U test. ****P < 0.0001.

Although CDK downregulation is observed only in early-stage CKD (4 weeks), most of the same cell cycle-related pathways remain active in kidneys with more advanced disease (Table 1). Notably, with disease progression, there is a significant shift towards increased activity of kinases involved in these selected pathways (Figure 3A).

**TABLE 1:** Cell cycle-related pathways.

	ID	Description	p.adjust
4 weeks	GO:0001558	Regulation of cell growth	1.70E-07
	GO:0045787	Positive regulation of cell cycle	5.66E-07
	GO:0016049	Cell growth	1.06E-06
	GO:0060284	Regulation of cell development	3.76E-05
	GO:0048146	Positive regulation of fibroblast proliferation	6.46E-05
	GO:0048144	Fibroblast proliferation	5.91E-04
	GO:0000082	G1/S transition of mitotic cell cycle	1.38E-03
	GO:0044843	Cell cycle G1/S phase transition	2.52E-03
	GO:0051783	Regulation of nuclear division	2.12E-02
	hsa04218	Cellular senescence	3.39E-02
	GO:1901992	Positive regulation of mitotic cell cycle phase transition	4.21E-02
12 weeks	GO:0045787	Positive regulation of cell cycle	5.38E-10
	GO:0016049	Ccell growth	1.98E-08
	GO:0001558	Regulation of cell growth	9.11E-08
	GO:0060284	Regulation of cell development	9.95E-07
	GO:0048146	Positive regulation of fibroblast proliferation	1.17E-03
	GO:0048144	Fibroblast proliferation	6.14E-03
	hsa04218	Cellular senescence	1.60E-02
	GO:0044843	Cell cycle G1/S phase transition	4.64E-02

Next, we examined the kinases that were significantly differentially activated within these selected pathways to identify those with the highest recurrence across pathways (Figures 3B,C). In the early-stage CKD, the most frequently repeated kinases are AKT1 and CDK4, which are both involved in 7 out of 11 listed pathways (Table 1; Figure 3B). Meanwhile, in late-stage CKD, CDKs are no longer differentially regulated; however, AKT1 remains highly represented, now accompanied by epidermal growth factor receptor (EGFR) and Abelson murine leukemia viral oncogene homolog 1 (ABL1). All three kinases are involved in 7 out of 7 of the listed pathways (Figure 3C). Overall, the same pathways are significantly different independent of the stage of CKD; however, the composition of active kinases shifted with disease progression. These changes highlight potential stage-specific kinase regulators that may contribute to CKD advancement.

### 3.4 CKD development affects kinases involved in key pathological processes

**FIGURE 4:**
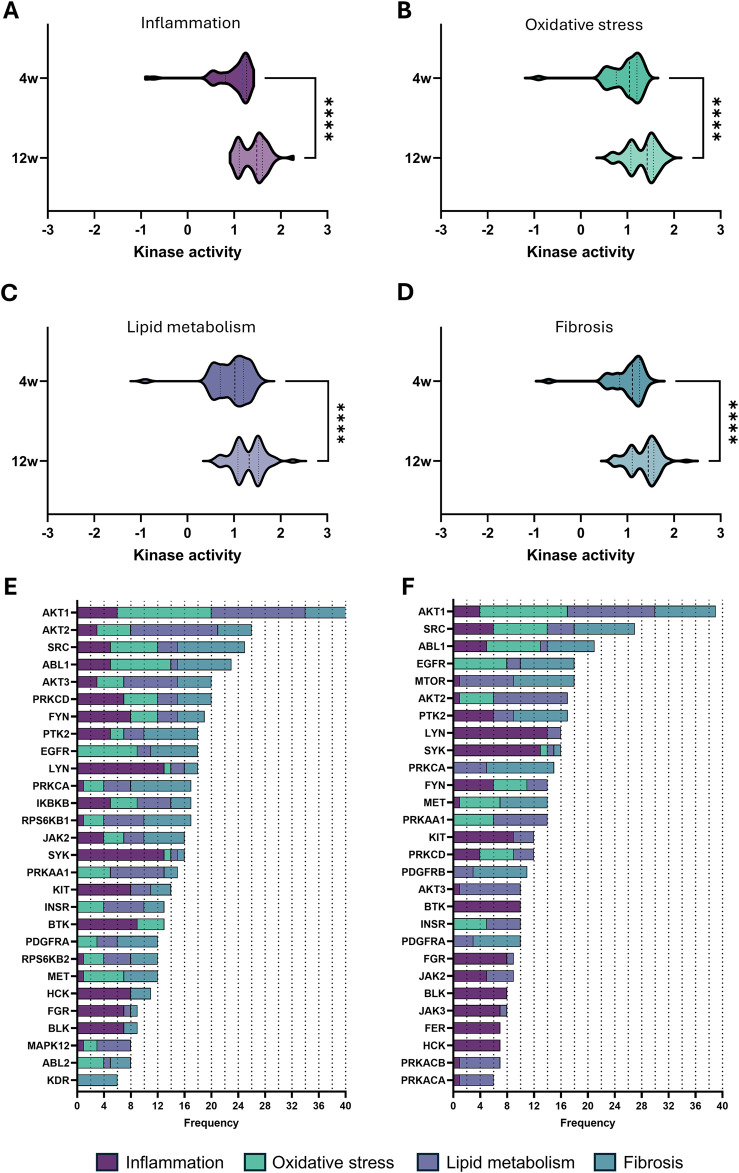
Kinase activity in the top 15 pathways related to major pathological processes in CKD. **(A–D)** Violin plot of kinase activity in pathways related to **(A)** inflammation, **(B)** oxidative stress, **(C)** lipid metabolism, and **(D)** fibrosis, based on the top 15 pathways identified (see Tables 2–5). Kinase activity is shown as the mean kinase statistic of each kinase involved in these pathways. **(E,F)** Bar graphs of kinase frequency among the top 15 pathways in each of the four pathological processes (See Tables 2–5 for all pathways; e.g., a frequency of 1 denotes that the kinase is involved in one pathway, while a frequency of 5 denotes that the kinase is involved in five distinctive pathways) after **(E)** 4 weeks and **(F)** 12 weeks of diet. Statistical significance was assessed by the Mann-Whitney U test. ****P < 0.0001.

Expanding on these apparent changes in cell cycle, we further focused on four key pathological processes involved in the progression of CKD (Yuan et al., 2022), namely, inflammation, oxidative stress, lipid metabolism, and fibrosis. For each process, the top 15 pathways ranked according to adjusted p-value were selected, excluding broad pathways that substantially overlapped across the selected processes as well as repeated pathways (Tables 2–5). Comparing kinase activity within these processes reveals a significant difference in kinase activity between the early-stage (4 weeks) and late-stage (12 weeks) CKD, with a general increase in activity at the more progressed CKD stage (Figures 4A–D). To identify the most influential kinases, we examined their presence across multiple pathways and ranked them by frequency of occurrence (Figures 4E,F). Similarly to the cell cycle analysis, AKT1 emerges as the most prominently involved kinase across the selected pathways. While some highly recurrent kinases, such as SRC, have already been studied in the field of CKD, others remain unexplored. A consistent trend was observed as certain kinases maintain high activity across both disease stages, whereas others show marked changes in both their activity and relevance as CKD progressed. These effects can also be directly appreciated by examining the various pathways (Table 2–5), which show considerable overlap between early and late stages, yet also stage-dependent shifts in the significance of the pathways. In contrast, some key pathways retain strong relevance throughout disease progression as evidenced by their significance (Table 2–5).

**TABLE 2:** Top 15 selected pathways related to inflammation.

	ID	Description	p.adjust
4 weeks	GO:0038093	Fc receptor signaling pathway	3.92E-20
	WP3929	Chemokine signaling pathway	8.16E-11
	GO:1903131	Mononuclear cell differentiation	9.56E-11
	GO:0070661	Leukocyte proliferation	1.18E-10
	GO:0006909	Phagocytosis	4.72E-10
	GO:0019221	Cytokine-mediated signaling pathway	5.82E-10
	GO:0002861	Regulation of inflammatory response to antigenic stimulus	3.63E-09
	hsa04660	T cell receptor signaling pathway	6.14E-09
	GO:0043299	Leukocyte degranulation	9.85E-09
	GO:0002279	Mast cell activation involved in immune response	1.12E-08
	GO:0002448	Mast cell mediated immunity	1.12E-08
	WP23	B cell receptor signaling pathway	1.35E-08
	GO:0046651	Lymphocyte proliferation	2.29E-08
	hsa04666	Fc gamma R-mediated phagocytosis	3.03E-08
	GO:0050900	Leukocyte migration	6.56E-08
12 weeks	GO:0038093	Fc receptor signaling pathway	1.93E-19
	GO:1903131	Mononuclear cell differentiation	8.75E-13
	GO:0006909	Phagocytosis	2.27E-11
	WP3929	Chemokine signaling pathway	4.72E-11
	GO:0070661	Leukocyte proliferation	5.58E-10
	GO:0002861	Regulation of inflammatory response to antigenic stimulus	7.62E-09
	GO:0050863	Regulation of T cell activation	7.68E-09
	GO:0043303	Mast cell degranulation	1.73E-08
	GO:0019221	Cytokine-mediated signaling pathway	2.08E-08
	GO:0043299	Leukocyte degranulation	2.13E-08
	GO:0002279	Mast cell activation involved in immune response	2.19E-08
	GO:0002448	Mast cell mediated immunity	2.19E-08
	GO:0050900	Leukocyte migration	3.38E-08
	GO:2000106	Regulation of leukocyte apoptotic process	4.65E-08
	GO:0070663	Regulation of leukocyte proliferation	4.66E-08

**TABLE 3:** Top 15 selected pathways related to oxidative stres**s**.

	ID	Description	p.adjust
4 weeks	hsa04012	ErbB signaling pathway	4.09E-19
	hsa04066	HIF-1 signaling pathway	6.34E-12
	GO:0000302	Response to reactive oxygen species	2.14E-10
	hsa04068	FoxO signaling pathway	7.19E-09
	GO:0034614	Cellular response to reactive oxygen species	1.40E-08
	GO:0006979	Response to oxidative stress	4.31E-08
	WP2324	AGE/RAGE pathway	5.46E-07
	hsa05208	Chemical carcinogenesis - reactive oxygen species	1.09E-05
	WP1403	AMP-activated protein kinase signaling	2.59E-05
	hsa04064	NF-kappa B signaling pathway	3.36E-05
	GO:0051341	Regulation of oxidoreductase activity	1.63E-03
	GO:0051353	Positive regulation of oxidoreductase activity	1.68E-03
	GO:1903201	Regulation of oxidative stress-induced cell death	3.81E-03
	GO:1902882	Regulation of response to oxidative stress	8.47E-03
	WP61	Notch signaling pathway	1.35E-02
12 weeks	hsa04012	ErbB signaling pathway	1.44E-19
	hsa04066	HIF-1 signaling pathway	1.22E-13
	GO:0000302	Response to reactive oxygen species	8.00E-10
	GO:0006979	Response to oxidative stress	2.52E-08
	GO:0034614	Cellular response to reactive oxygen species	3.54E-08
	WP2324	AGE/RAGE pathway	1.13E-06
	hsa04068	FoxO signaling pathway	1.46E-06
	WP4357	NRF2-ARE regulation	2.51E-06
	WP1403	AMP-activated protein kinase signaling	9.54E-05
	hsa05208	Chemical carcinogenesis - reactive oxygen species	1.44E-04
	hsa04064	NF-kappa B signaling pathway	4.50E-04
	GO:0036294	Cellular response to decreased oxygen levels	1.69E-03
	GO:0051353	Positive regulation of oxidoreductase activity	2.28E-03
	GO:1903201	Regulation of oxidative stress-induced cell death	5.27E-03
	GO:1902882	Regulation of response to oxidative stress	1.15E-02

**TABLE 4:** Top 15 selected pathways related to lipid metabolis**m**.

	ID	Description	p.adjust
4 weeks	WP481	Insulin signaling	2.73E-14
	GO:0045834	Positive regulation of lipid metabolic process	3.72E-13
	GO:0010517	Regulation of phospholipase activity	3.92E-12
	hsa04931	Insulin resistance	8.59E-10
	hsa05417	Lipid and atherosclerosis	3.68E-08
	hsa04072	Phospholipase D signaling pathway	2.12E-07
	hsa04923	Regulation of lipolysis in adipocytes	5.03E-07
	WP2034	Leptin signaling pathway	1.53E-06
	WP3915	Angiopoietin-like protein 8 regulatory pathway	2.29E-06
	WP3965	Lipid metabolism pathway	5.21E-06
	hsa04920	Adipocytokine signaling pathway	2.09E-05
	WP1403	AMP-activated protein kinase signaling	2.59E-05
	hsa04071	Sphingolipid signaling pathway	2.81E-04
	GO:0044539	long-chain fatty acid import into cell	6.95E-04
	WP26	S1P receptor signal transduction	7.63E-04
12 weeks	GO:0045834	positive regulation of lipid metabolic process	1.18E-17
	GO:0010517	Regulation of phospholipase activity	1.19E-14
	hsa04931	Insulin resistance	3.31E-09
	WP2374	Oncostatin M signaling pathway	5.77E-09
	hsa04072	Phospholipase D signaling pathway	9.38E-08
	hsa05417	Lipid and atherosclerosis	9.03E-07
	hsa04923	Regulation of lipolysis in adipocytes	1.20E-06
	hsa04922	Glucagon signaling pathway	1.84E-06
	hsa04910	Insulin signaling pathway	2.17E-06
	hsa04911	Insulin secretion	2.44E-06
	WP2034	Leptin signaling pathway	3.22E-06
	hsa04071	Sphingolipid signaling pathway	4.23E-06
	WP3965	Lipid metabolism pathway	8.96E-06
	hsa04920	Adipocytokine signaling pathway	4.23E-05
	WP1403	AMP-activated protein kinase signaling	9.54E-05

**TABLE 5:** Top 15 selected pathways related to fibrosis.

	ID	Description	p.adjust
4 weeks	WP4540	Hippo signaling regulation pathways	9.88E-24
	hsa01521	EGFR tyrosine kinase inhibitor resistance	4.60E-21
	hsa04012	ErbB signaling pathway	4.09E-19
	WP306	Focal adhesion	1.14E-14
	GO:0048013	Ephrin receptor signaling pathway	3.10E-13
	WP3932	Focal adhesion: PI3K-Akt-mTOR-signaling pathway	7.35E-13
	WP437	EGF/EGFR signaling pathway	6.18E-12
	GO:0022407	Regulation of cell-cell adhesion	2.77E-11
	GO:0031532	Actin cytoskeleton reorganization	4.73E-11
	WP4566	Translation inhibitors in chronically activated PDGFRA cells	5.96E-11
	GO:0034329	Cell junction assembly	4.74E-10
	GO:0032970	Regulation of actin filament-based process	1.21E-08
	WP2332	Interleukin-11 signaling pathway	3.23E-07
	GO:0045787	Positive regulation of cell cycle	5.66E-07
	GO:0031589	Cell-substrate adhesion	1.60E-06
12 weeks	WP4540	Hippo signaling regulation pathways	3.84E-32
	hsa01521	EGFR tyrosine kinase inhibitor resistance	3.60E-20
	hsa04012	ErbB signaling pathway	1.44E-19
	WP4541	Hippo-Merlin signaling dysregulation	1.03E-15
	GO:0048013	Ephrin receptor signaling pathway	2.15E-14
	WP437	EGF/EGFR signaling pathway	2.97E-13
	GO:0032970	Regulation of actin filament-based process	1.30E-12
	GO:0032956	Regulation of actin cytoskeleton organization	1.78E-12
	WP4566	Translation inhibitors in chronically activated PDGFRA cells	7.39E-12
	GO:0022407	Regulation of cell-cell adhesion	2.01E-11
	WP3932	Focal adhesion: PI3K-Akt-mTOR-signaling pathway	5.01E-11
	hsa04510	Focal adhesion	1.98E-10
	GO:0045787	Positive regulation of cell cycle	5.38E-10
	GO:0034329	Cell junction assembly	2.49E-09
	WP2374	Oncostatin M signaling pathway	5.77E-09

## 4 Discussion

CKD, particularly when accompanied by cardiovascular complications, remains a major global health burden, yet the underlying mechanisms are still not fully understood. To gain more insights into these mechanisms, we deployed an unbiased kinomics approach on the renal cortex of hyperlipidemic mice with or without CKD. Thereby, we could identify numerous kinases that are differentially regulated in early- or late-stage CKD development. These kinases were related to several key pathological processes of CKD, including inflammation, oxidative stress, and fibrosis, as well as lipid metabolism pathways.

Interestingly, one of the highly upregulated kinases that was also most prominent in the selected pathological processes is Akt1, while its family members, Akt2 and Akt3, were also prominently represented in these processes. This observation also validates our study, as it has already been well described that Akt signaling plays an essential role in CKD (Lan and Du, 2015; Wang H. et al., 2024). For instance, genetic deletion of *Akt1* in a mouse model that mimics the transition of acute kidney injury to CKD resulted in an attenuation of tubulointerstitial fibrosis and tubular apoptosis (Kim et al., 2023). Another kinase group strongly associated with CKD is the Src family kinases, consisting of eight family members: Src, Fyn, Yes1, Lyn, Fgr, Hck, Blk, and Lck (Wang and Zhuang, 2017). Particularly, Src, Fyn, and Hck have already been widely studied in CKD and have been identified as critical mediators of renal fibrosis. For example, inactivation of Src in a mouse model of renal interstitial fibrosis resulted in reduced renal fibroblast activation and attenuated extracellular matrix protein deposition (Yan et al., 2016). Furthermore, Fyn-deficient mice have also been shown to exhibit attenuated renal fibrosis upon ureteral obstruction (Seo et al., 2016), further demonstrating the importance of Src family kinases in renal diseases. Strikingly, all eight family members were significantly upregulated in both the early- and late-stage CKD in our study, again validating our results. Interestingly, the other Src family kinases (Blk, Fgr, Hck, Lck and Lyn) are still relatively unexplored in CKD. However, we observed similar upregulation of these kinases upon CKD development, compared to, for example, Src or Fyn. Therefore, these may also be interesting kinases to further study in this context, especially since many Scr family kinases have redundant roles (Wang and Zhuang, 2017).

Examining additional kinases involved in the selected pathological pathways revealed several that have already been linked to CKD in the literature, although less widely studied so far. For example, ABL has been demonstrated to drive fibroblast activation and differentiation during renal fibrosis, through the regulation of RACK1 stabilization (Bao et al., 2024). Furthermore, PRKCD (PKCδ), is upregulated in fibrotic kidneys, while the PKCδ inhibitor, rottlerin, alleviated both renal fibrosis and inflammation (Wang D. et al., 2024). PTK2, also known as FAK, is another kinase that has already been shown to be upregulated during the transition from acute kidney injury to CKD. In line with this, mice that have a renal collecting duct-specific *Ptk2* deficiency demonstrated reduced oxidative stress and attenuated renal fibrosis (Gao et al., 2025).

Due to the unbiased approach of our study, we were also able to identify kinases that have not yet been described in the context of CKD. For example, BTK, which is considered an essential regulator of immunoregulation (Weber et al., 2017), was also identified as a kinase that is increased upon CKD development in our mice and involved in the selected key pathways. Although some studies are showing the involvement of BTK in diabetic nephropathy (Zhao et al., 2021), hemolytic-uremic syndrome (Kroller et al., 2023), and lupus nephritis (Chalmers et al., 2018), the role of BTK in CKD has not yet been explored. Another notable kinase, which was not involved in the selected pathways, yet was still highly upregulated upon CKD development, is PSKH1. Although PSKH1 has recently been identified as a crucial factor in kidney development, no studies have yet elucidated its potential pathological role in kidney disease. Based on our results, this would be an interesting kinase to study in the future. Therefore, our unbiased kinome profiling provides the opportunity to identify novel mediators involved in CKD development and is thus a valuable source for future lead identification.

Another interesting observation of our unbiased kinomics analysis is that CDKs are significantly less active in the early phase of CKD development, while this inhibition is abolished upon disease progression. CDK kinases play a crucial role in the regulation of the cell cycle, and this observation aligns with previous studies showing that a cell cycle arrest at G2 occurs, particularly in tubular epithelial cells, during the initial stages of kidney damage. At the same time, this is not the case anymore at a later stage (Koyano et al., 2019; Lovisa et al., 2015). This cell cycle arrest is dependent on the CDK inhibitor p21, whose presence appears protective, as *p21*-deficient mice exacerbate the progression of renal fibrosis (Koyano et al., 2019), demonstrating its importance in disease development. Several studies evaluate the effects of CDK inhibitors on CKD development (Pellarin et al., 2025). For example, the blockage of CDK4/6 in mice has been shown to improve renal function and reduce tubular injury and fibrosis (Osaki et al., 2022). Paradoxically, selective deletion of cyclin D1, which complexes with CDK4/6, was shown to increase tubular injury (Osaki et al., 2022), suggesting that potential therapeutic strategies should be rather specific and prioritize the inhibition of CDK4/6. Overall, CDKs play a key role in the development of CKD; however, based on our results, supported by literature, their role is very stage-dependent.

Overall, this study identified key and novel kinases that are regulated during CKD development in hyperlipidemic mice. While validating several key kinases, the deployed unbiased kinomics approach also identified several novel kinases that so far have not yet been described to play a role in CKD development. Therefore, these results are a valuable source for future studies to investigate further whether these novel identified kinases also play a causal role in CKD development.

## Data Availability

The raw data supporting the conclusions of this article will be made available by the authors, without undue reservation.
