# Supplementary figures and images for: Unbiased kinome profiling identifies key and novel mediators of chronic kidney disease in hyperlipidemic mice
Source: Front Physiol. 2025 Oct 2;16:1684982. doi: 10.3389/fphys.2025.1684982 (PMC12528016; doi:10.3389/fphys.2025.1684982)

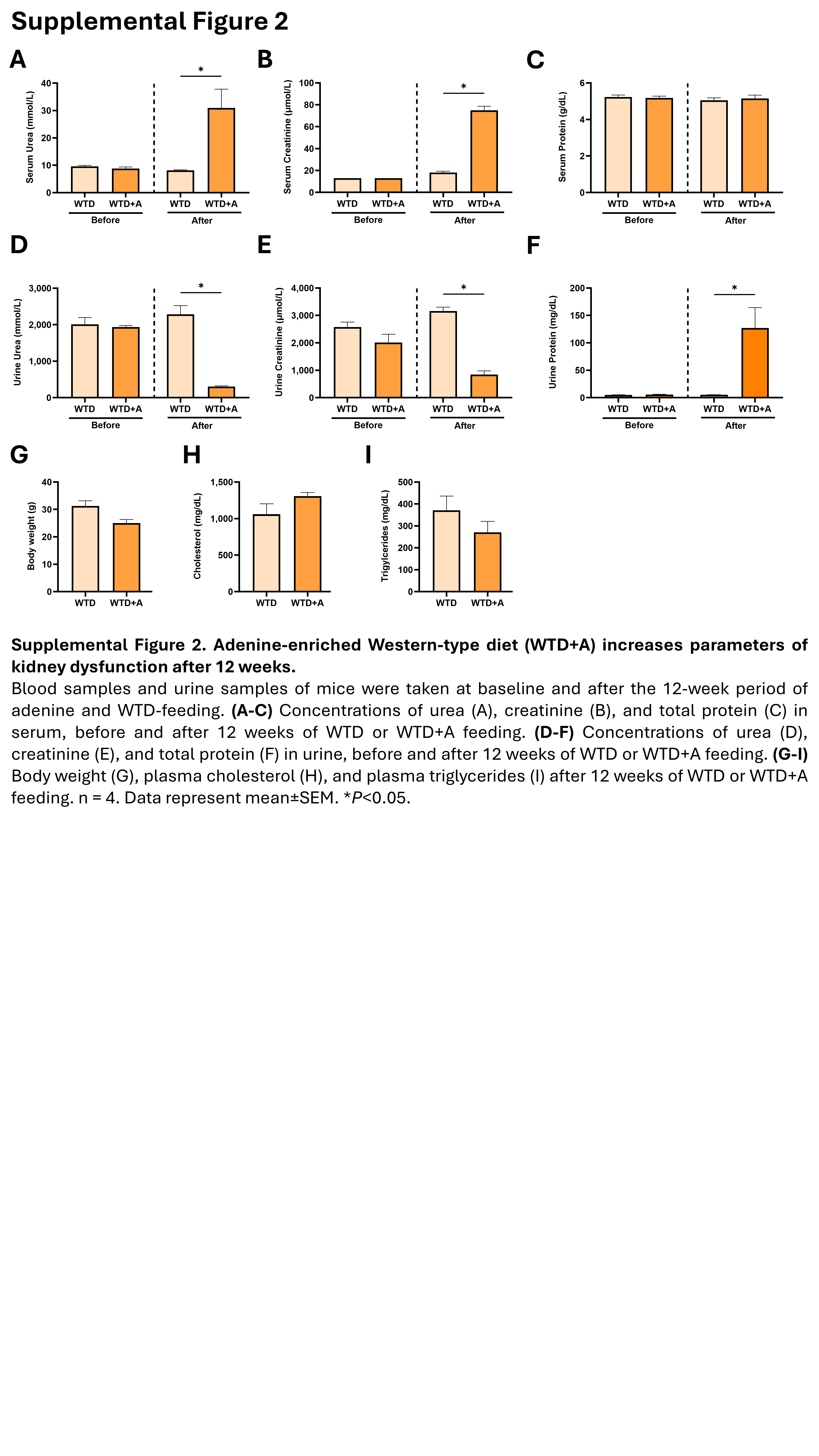

Supplement: Supplementary file 3 [file Image2.tif]

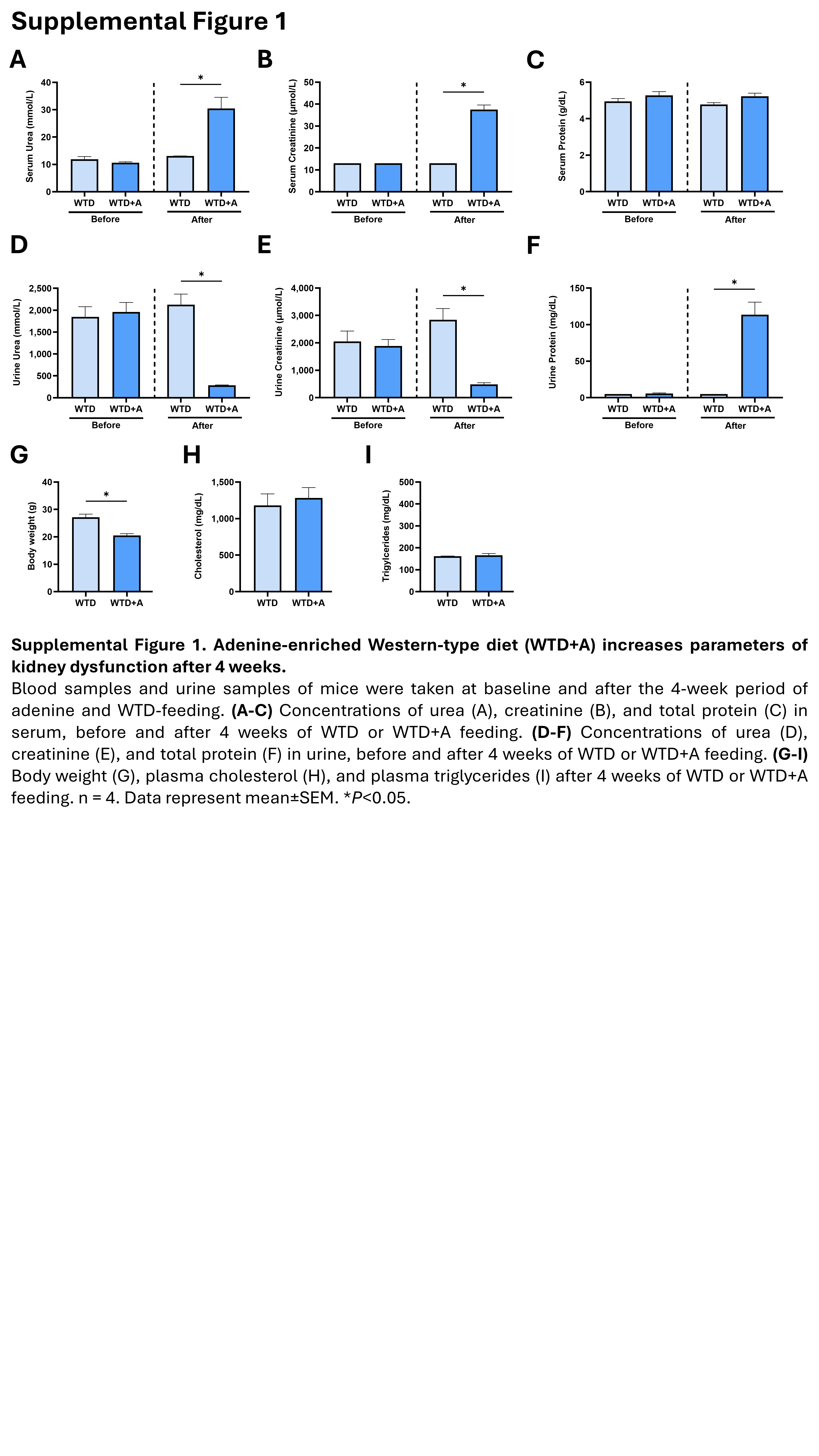

Supplement: Supplementary file 4 [file Image1.tif]
